# Common cytokine receptor gamma chain family cytokines activate MAPK, PI3K, and JAK/STAT pathways in microglia to influence Alzheimer’s Disease

**DOI:** 10.3389/fnmol.2024.1441691

**Published:** 2024-09-11

**Authors:** Hannah Zuppe, Erin Reed

**Affiliations:** ^1^School of Biomedical Sciences, Kent State University, Kent, OH, United States; ^2^Department of Pharmaceutical Sciences, Northeast Ohio Medical University, Rootstown, OH, United States

**Keywords:** Alzheimer’s Disease, neuroinflammation, microglia, interleukins, cellular signaling

## Abstract

Dementia is an umbrella term used to describe deterioration of cognitive function. It is the seventh leading cause of death and is one of the major causes of dependence among older people globally. Alzheimer’s Disease (AD) contributes to approximately 60–70% of dementia cases and is characterized by the accumulation of amyloid plaques and tau tangles in the brain. Neuroinflammation is now widely accepted as another disease hallmark, playing a role in both the response to and the perpetuation of disease processes. Microglia are brain-resident immune cells that are initially effective at clearing amyloid plaques but contribute to the damaging inflammatory milieu of the brain as disease progresses. Circulating peripheral immune cells contribute to this inflammatory environment through cytokine secretion, creating a positive feedback loop with the microglia. One group of these peripherally derived cytokines acting on microglia is the common cytokine receptor γ chain family. These cytokines bind heterodimer receptors to activate three major signaling pathways: MAPK, PI3K, and JAK/STAT. This perspective will look at the mechanisms of these three pathways in microglia and highlight the future directions of this research and potential therapeutics.

## 1 Introduction

Alzheimer’s Disease (AD) comprises approximately 60–70% of dementia cases and can be sub-categorized based on age of onset and inheritance pattern (WHO, 2023). Early-and late-onset forms of AD are defined as whether disease manifested before or after age 65, respectively (Reitz et al., 2020). Mendelian inheritance of AD is characterized by fully penetrant autosomal dominant inheritance of genes like mutated amyloid precursor protein (APP), presenilin 1 (PSEN1), and presenilin (PSEN2), though it has also been used to describe AD patients with a family history of the disease (Reitz et al., 2020). Sporadic inheritance is characterized by inheritance patterns that are not obviously autosomal dominant or have a highly variable age at onset (Reitz et al., 2020). AD can be clinically described as “positive” brain lesions of amyloid plaques, tau tangles, and glial inflammatory responses and “negative” brain lesions of neuronal and synaptic loss (Serrano-Pozo et al., 2011). These “negative” lesions, especially synaptic loss, closely parallel cognitive decline (Serrano-Pozo et al., 2011). Neuroinflammation, localized to “positive” lesions, plays a significant role in both the response to and the perpetuation of disease processes, thereby acting as a “double-edged sword” (Swanson et al., 2018).

Microglia are brain-resident immune cells that play pivotal roles in neuroinflammation in AD (O’Connor and Nissen, 2023). In early disease stages, microglia are efficient at clearing amyloid plaque; however, as disease progresses, microglia transition from an “acute” to a “chronic” inflammatory phenotype often referred to as neurodegenerative microglia (MGnD) or disease-associated microglia (DAM) that are less effective at amyloid removal (O’Connor and Nissen, 2023; Kinney et al., 2018; Poppell et al., 2023; Ocañas et al., 2023). These MGnD/DAM microglia are thought to be responsible for damaging inflammation resulting in neuronal death (O’Connor and Nissen, 2023; Kinney et al., 2018; Poppell et al., 2023). Circulating peripheral immune cells contribute to this damaging inflammatory environment through cytokine secretion, creating a positive feedback loop with the microglia.

One group of these peripherally derived cytokines that act on microglia is the common cytokine receptor γ chain family, which includes the interleukins IL-2, IL-4, IL-7, IL-9, IL-15, and IL-21. These cytokines bind heterodimer receptors to activate three major signaling pathways: mitogen-activated protein kinase (MAPK), phosphatidylinositol 3-kinase (PI3K), and Janus kinase/signal transducers and activators of transcription (JAK/STAT). This perspective focuses on the effect these cytokine receptors have on microglia, as they are upstream of signaling pathways implicated in AD-related neuroinflammation and occur in the immune cell considered most responsible for the inflammatory response (Table 1).

**Table 1:** Effect of common cytokine receptor γ chain cytokines on microglia and AD overall.

Cytokine	Binding receptors	Effect on microglia	Anomalies in AD	References
IL-2	Heterotrimer of: IL-2Rα/CD25 IL-2/IL-15Rβ Common γ-chain/IL-2Rγ	Activation of JAK/STAT	Downregulated Treatment increased T_reg_ levels, astrocyte recruitment, synaptic plasticity, and rescued spine density	Sarapultsev et al. (2023), Yshii et al. (2022), Owen et al. (2018), Hanisch and Quirion (1995), Alves et al. (2017)
IL-4	Type 1 (specific): IL-4Rα Common γ-chain/IL-2Rγ Type 2 (IL-4, IL-13): IL-4Rα IL-13Rα	Downregulation of TLRs Inhibition of NF-κB Upregulation of PPARγ genes “M2” phenotype Activation of JAK/STAT	Treatment resulted in “M2” microglia, decreased Aβ deposition	Sarapultsev et al. (2023), Latta et al. (2015), Dionisio-Santos et al. (2020), Zuiderwijk-Sick et al. (2021), Zhao et al. (2015), Taipa et al. (2019)
IL-7	Heterodimer of: IL-7Rα Common γ-chain/IL-2Rγ	Activation of JAK/STAT	Upregulation in the CSF	Sarapultsev et al. (2023), Taipa et al. (2019)
IL-9	Heterodimer of: IL-9R Common γ-chain/IL-2Rγ	IL-9R is highly expressed Activation of JAK/STAT	Upregulation in African Americans but not Caucasians	Sarapultsev et al. (2023), Taipa et al. (2019), Ding et al. (2015), Wharton et al. (2019)
IL-15	Heterotrimer of: IL-15Rα IL-2/IL-15Rβ Common γ-chain/IL-2R γ	Astrocyte-microglia crosstalk Blockade inhibits MAPK and NFκB signaling Activation of JAK/STAT	Conflicting inflammatory and anti-inflammatory effects	Sarapultsev et al. (2023), Pan et al. (2013), Shi et al. (2020), Hanisch et al. (1997)
IL-21	Heterodimer of: IL-21R Common γ-chain/IL-2R γ	Upregulation of inflammation Activation of JAK/STAT	Increased of IL-21 and IL-21R Increased Aβ plaque Alterations to lymphocyte and macrophage levels Increased IL-6 and IL-18	Sarapultsev et al. (2023), Agrawal et al. (2022)

Il-2 is very low during AD, so analogs that could pass through the blood brain barrier (BBB) could be potential therapeutics. IL-4 has also been gaining traction as a possible therapeutic target for AD due to its ability to polarize microglia toward an anti-inflammatory phenotype by downregulating TLRs and NF-κB signaling and upregulating PPARγ genes. It is unclear what effect IL-7 has on microglia and AD overall, though it is known to be upregulated in the CSF of AD patients. The IL-9R is highly expressed on microglia and is upregulated in autoimmune disorders. Its role in AD seems to be race dependent as it is upregulated in African Americans but not in Caucasians. IL-15 is generally grouped with IL-2 in terms of microglia signaling pathways due to the large overlap in receptors. Its role in AD is conflicting. IL-21 has a highly inflammatory effect on microglia and peripheral immune cells though the exact mechanisms are unclear.

Modulation of these three signaling pathways to treat AD by reducing the highly inflammatory milieu of the brain has been gaining traction in the field. These pathways have been successfully targeted in other inflammatory disorders, so it is very appealing to repurpose relatively safe and inexpensive treatments for AD patients. The leading issue with these reutilizations is that the drugs often have low Blood–Brain Barrier (BBB) permeability. There are currently several clinical trials addressing the safety and efficacy of these inhibitors after promising results in pre-clinical studies.

## 2 MAPK signaling pathway

The MAPK signaling pathway has three major components: the MAPK Kinase Kinase (MAPKKK), the MAPK Kinase (MAPKK), and MAPK. MAPKKK is usually phosphorylated by a protein kinase downstream of the cytokine receptor. MAPKKK phosphorylates MAPKK, which in turn phosphorylates MAPK. MAP Kinases are the third major kinase activated in the MAPK signaling pathway and are well-known for their roles in cellular proliferation, differentiation, or death. These kinases can be grouped into three families: ERK (extracellular-signal-regulated kinases), JNK (Jun amino-terminal kinases), and p38/SAPK (stress-activated protein kinases). We refer the reader to the excellent review by Morrison for detailed descriptions of the signaling mechanisms in these pathways (Morrison, 2012).

Depending on the specific kinases involved, MAPK signaling results in cell growth, survival, differentiation, inflammation, or apoptosis. Classic ERK signaling begins with a mitogen or growth factor and results primarily in cellular growth and differentiation (Morrison, 2012). JNK signaling is typically activated by environmental stress like oxidative stress, or inflammatory cytokines, but can also occur in response to growth factors, resulting in inflammation and metabolic changes (Morrison, 2012). It may also play a role in apoptosis, but whether it is apoptotic, anti-apoptotic, or uninvolved is unresolved (Morrison, 2012; Lin, 2003). P38 signaling, like JNK, is typically activated by environmental stress or inflammatory cytokines primarily resulting in inflammation, differentiation, apoptosis, or cell cycle regulation (Morrison, 2012). In AD, ERK, JNK, and p38 are upregulated in vulnerable neurons indicating involvement in AD pathogenesis.

Less is known about the role of MAPK in microglia. A recent study has indicated p-ERK expression is linked to microglia adopting a “DAM” phenotype and damaging inflammation in both an amyloid mouse model (5xFAD) and post-mortem patient samples (Chen et al., 2021). ERK signaling is activated downstream of the RTKs CSF1R and MERTK in homeostatic microglia, while it is activated by AXL and FLT1 in DAMs (Chen et al., 2021). It is critical in the regulation of IFNγ-mediated inflammation (Chen et al., 2021). Furthermore, ERK is upstream of several DAM and human AD risk genes such as TREM2, Tyrobp, Bin1, Cd33, Trem2, and Cnn2 (Chen et al., 2021). Analysis of post-mortem human brains found that ERK1 and ERK2 were the only MAPK signaling proteins with increased protein expression and positive associations with neuropathological grade (Chen et al., 2021).

JNK is mostly activated in AD by the environmental stress of the APP cleavage product amyloid-β (Aβ) (Solas et al., 2023). When Aβ was given to wild type mice, p-JNK levels were significantly elevated (Solas et al., 2023). In a follow-up experiment, JNK3 overexpression via a viral vector caused cognitive deficiencies and tau misfolding in Tg2576 mice but did not impact amyloid pathology (Solas et al., 2023). Taken together, this suggests that JNK signaling is in response to Aβ but affects tau pathology (Solas et al., 2023). Whether JNK activation precedes or is in response to amyloid plaque in AD patients is under debate. p-JNK was significantly increased in the post-mortem brains of AD patients, but it was also upregulated in non-AD related dementia patients (Solas et al., 2023). However, there was a co-localization between p-JNK and Aβ (Solas et al., 2023).

P38 is mainly expressed by brain resident immune cells (glia) during neurodegeneration though previous research has mostly focused on its role in neurons mostly due to its ability to phosphorylate tau and consequently worsen pathology (Perea et al., 2022; Asih et al., 2020). In a tau mouse model (P301S), p38 activation increased during aging primarily in hippocampal microglia (Perea et al., 2022). Interestingly, this model produced p38^low^ microglia that seemed to have a neuroprotective effect in this context (Perea et al., 2022).

All three families of MAP kinases stimulate inflammatory cytokine production in microglia. Microglia treated with Aβ or APP showed activation of the three MAPKs (Kim et al., 2004; Bodles and Barger, 2005). Interestingly, only JNK and p38 inhibitors reduced both nitrite and nitric oxide synthase (iNOS) accumulation, indicating only JNK and p38 are involved in microglial activation that results in neuronal damage (Bodles and Barger, 2005). This has also been seen in a microglial-neuronal co-culture experiment where inhibitors to JNK and p38, but not ERK, prevented neuronal death (Xie et al., 2004). Preliminary experiments implicate Toll-Like Receptor 4 (TLR4) upregulation on microglia resulting in JNK and p38 mediated neuronal damage though there has not been follow-up (Gaikwad et al., 2015).

Two p38 inhibitors, MW150 and Neflamapimod, are currently in clinical trials for AD treatment. Both were developed to mitigate the off-target effects on the heart and liver often seen with first generation p38α inhibitors caused by non-p38 kinases being inhibited at higher concentrations (Libby and Everett, 2019; Hammaker and Firestein, 2010). MW150 is a p38α inhibitor developed by Neurokine Therapeutics that is currently in a phase 2a randomized double-blind, placebo-controlled, study, in mild-to-moderate AD patients though results have not yet been released. Fourteen-day treatment with MW150 improved symptoms across animal models though the specific effects differed. In one study, improved synaptic function and cognition was observed in both older and younger animals (Rutigliano et al., 2018). However, in another study, treatment did not affect plaque load or immune cells markers but did decrease inflammatory cytokine levels (Zhou et al., 2017). Another study found that MW150 improved hippocampal-dependent memory in two AD animal models while also being highly specific to p38α (Roy et al., 2015). It is hoped that the specificity and robustness of MW150 treatment in animal models will translate into the same response in patients.

Neflamapimod, developed by EIP Pharma, is also in phase 2 studies to test efficacy. In an open-label clinical study, patients had improved immediate and delayed recall compared to baseline, correlating with drug plasma levels (Scheltens et al., 2018). It was also able to cross the BBB and decrease TNF-α and IL-8 in the CSF. However, it should be noted that this study was not placebo controlled (Scheltens et al., 2018; Alam et al., 2017). It appears that Neflamapimod may be better suited to treating dementia caused by Lewy Bodies than dementia caused by AD, as it is currently in a Phase 2b (hypothesis-testing), multi-center, randomized, double-blind, placebo-controlled study for treating dementia with Lewy Bodies. It is possible that Neflamapimod could be revisited as a treatment for AD, especially if the current clinical trial is successful.

## 3 PI3K signaling pathway

The PI3K pathway is most known for regulating cell survival though it also functions in cellular proliferation, growth, differentiation, and motility. PI3K is activated by kinases associated with the cytokine receptor. It then phosphorylates PIP2 to PIP3. PIP3 goes on to phosphorylate AKT, either directly or by activating mTORC2 or PDK1 that then activates AKT. AKT can act on numerous substrates but is most well-known for activating mTORC1, which is notably responsible for mRNA translation, autophagy, and mitochondrial functions (Sun et al., 2020). The pathway is generally inhibited by dephosphorylation of PIP3 into PIP2 by PTEN or dephosphorylation of AKT. The PI3K pathway in microglia is closely associated with many other signaling pathways, making it an attractive therapeutic target. Interestingly, PI3K and ERK signaling exhibit mutual inhibition (Manning and Toker, 2017).

While in a healthy brain, the PI3K pathway is associated with neuronal development and plasticity, in an AD brain, PI3K is associated with neuronal toxicity and inflammatory phenotypes of astrocytes and microglia. The PI3K/AKT pathway becomes dysregulated in AD, due to mutations that seemingly suppress its negative regulation (Chu et al., 2021; Tsai et al., 2021; Yoshino et al., 2017; Yao et al., 2019). Clinically, this represents a risk factor for late-onset AD and is linked to disease-related neuropathology (Chu et al., 2021; Efthymiou and Goate, 2017).

This pathway is generally accepted as the central regulator of microglial activation in response to stimulation. Most of this signaling is downstream of the TLR4 receptor, which is increased in AD (Chu et al., 2021; Fang et al., 2017). IL-4 can also activate PI3K signaling, but it is reduced in AD models (Gadani et al., 2012). Activation of PI3K signaling notably results in microglia secreting pro-inflammatory factors and can be further exacerbated by mitochondrial dysfunction increasing ROS and iNOS species that then activate NF-κB (Chu et al., 2021; Hoogland et al., 2018; Willis et al., 2020; Tönnies and Trushina, 2017; Nakajima and Kohsaka, 2001).

AKT activity may be neuroprotective in certain situations, especially in younger models as AKT exhibits age-related shifts in signaling (Razani et al., 2021). Microglia adopt a more chronic inflammatory phenotype that has reduced phagocytic capabilities during aging (Njie et al., 2012), though whether this results in or is an effect of AKT signaling is unclear. The role of mTORC proteins has also been inconsistent in AD mouse models. While the early use of mTORC inhibitors like rapamycin have decreased AD disease hallmarks, these inhibitors exacerbated damage in later disease stages, potentially due to the inhibition of mTORC1 interacting with TREM2, which improves amyloid plaque clearance (Razani et al., 2021; Shi et al., 2022). However, it is unclear whether this is a distinct signaling pathway from the classic inflammatory one.

Modulation of the PI3K pathway to treat AD has been gaining traction because it could affect both neuronal and glia cells. However, only rapamycin, an mTORC1 inhibitor, is currently in a phase 2 clinical trial. It should be noted that rapamycin is most effective at early disease stages and can have side effects (Razani et al., 2021; Majumder et al., 2011; Carosi and Sargeant, 2019). For those reasons, a second generation of mTORC inhibitors is being developed that can act on both mTORC1 and mTORC2, and more recently, dual mTORC and PI3K inhibitors have also been developed (Razani et al., 2021; Dowling et al., 2010; Benjamin et al., 2011; Zaytseva et al., 2012). One dual inhibitor, NVPBEZ235, was found to improve memory impairment and microglia activation in an AD mouse model (Razani et al., 2021; Bellozi et al., 2016; Bellozi et al., 2019).

## 4 JAK/STAT signaling pathway

The JAK/STAT pathway is the backbone of many intracellular signaling processes due to rapid membrane-to-nucleus signaling. Unlike MAPK and PI3K signaling, for which the classical signaling occurs in the cytoplasm, JAK/STAT has a nuclear signaling component. The JAK kinases are non-covalently associated with receptors and are responsible for mediating tyrosine phosphorylation of receptors and recruiting STAT proteins. Tyrosine-phosphorylated STATs dimerize and are transported to the nucleus to regulate specific genes. JAK/STAT is seemingly the most prevalent of the three pathways in AD, as is responsible for the dysregulation of immune responses (Miao et al., 2023). It has been hypothesized that JAK inhibitors could be repurposed to help treat AD patients.

Mammals have four types of JAK and seven types of STAT, where the cytokine or growth factor initiating the cascade determines JAK and STAT protein activation (Table 2) (Sarapultsev et al., 2023). During neuroinflammation, these JAK/STAT signaling cascades typically result in microglia adopting an inflammatory phenotype. The notable exception to this is IL-4 activation of STAT6, mediating the re-polarization of microglia to an anti-inflammatory phenotype (Sarapultsev et al., 2023; He et al., 2020).

**Table 2:** JAK/STAT pathways activated by common cytokine receptor γ chain family cytokines and their notable effects in microglia.

Common cytokine receptor γ chain family cytokine	JAK	STAT	Notable effects in microglial inflammation	References
IL-2	JAK1 JAK2 JAK3	STAT1 STAT2 STAT3 STAT4 STAT5	JAK1/STAT1/NF-κB results in microglial-mediated neuroinflammation JAK2/STAT1 results in inflammatory microglial phenotype STAT5/NF-κB results in inflammatory microglial phenotype	Sarapultsev et al. (2023), Li et al. (2022), Zang et al. (2022), Pu et al. (2022)
IL-4	JAK1 JAK3	STAT6	IL-4/JAK1/STAT6 results in microglia re-polarization from “M1” to “M2” Inhibition of IL-4Rα/JAK1 and JAK3/STAT6 results in “M1” microglial phenotype	Sarapultsev et al. (2023), He et al. (2020), Im et al. (2020)
IL-7 IL-9 IL-15 IL-21	JAK1 JAK3	STAT1, STAT3 activated by JAK1 STAT5 activated by either JAK1 or JAK3	JAK1/STAT1/NF-κB results in microglial-mediated neuroinflammation STAT5/NF-κB results in inflammatory microglial phenotype	Sarapultsev et al. (2023), Li et al. (2022), Pu et al. (2022)

This class of cytokines typically activates JAK1 or JAK3 that then activate STAT1, STAT3, or STAT5. The STAT dimers can then act on genes like NF-κB to promote an inflammatory phenotype in microglia. The notable exception to this is IL-4 that results in the activation of STAT6, which promotes an anti-inflammatory phenotype in microglia. Table based on those found in Sarapultsev et al. (2023).

Over-activation of the JAK/STAT pathway in AD is typically associated with STAT3, likely due to STAT3 phosphorylation being increased in the hippocampus of both mouse models and post-mortem brains (Millot et al., 2020). In addition, STAT3 may be a transcriptional regulator of BACE1, an important enzyme in Aβ production (Millot et al., 2020). STAT3 is known to be involved in microglia adopting a chronic inflammatory phenotype in response to Aβ and its inhibition significantly decreased microglia activation (Sarapultsev et al., 2023; Millot et al., 2020; Przanowski et al., 2014). This finding has been corroborated in an AD mouse model (APP/PS1) where STAT3 deficiency promoted Aβ phagocytosis by microglia (Doty et al., 2016). Interestingly, STAT3 signaling in response to Aβ shows time-dependent modifications. First, acetylated STAT3 levels increase, the consequence of which is currently unclear (Eufemi et al., 2015). This is followed by increased levels of phosphorylated STAT3, shifting the microglial proteome to one of chronic activation (Eufemi et al., 2015). Levels of 14–3-3ε, a known marker of Aβ-activated microglia, are eventually increased (Eufemi et al., 2015).

It has often been hypothesized that JAK/STAT inhibitors could be re-purposed to treat AD. Since these inhibitors are relatively safe and inexpensive, their re-utilization in AD to suppress neuroinflammation would greatly improve current treatment plans. However, low BBB permeability in most FDA-approved JAK/STAT inhibitors remains an issue. Hydroxychloroquine is a STAT3 inhibitor that is currently approved to treat rheumatoid arthritis and systemic lupus erythematosus. In rheumatoid arthritis patients, hydroxychloroquine treatment was associated with a lower risk of AD incident compared to methotrexate treatment, accounting for biases like informative censoring, reverse causality, and outcome misclassification (Varma et al., 2023). This was followed by analysis in an AD mouse model (APP/PS1) that found hydroxychloroquine is a potential treatment for AD by inactivating STAT3 in neurons, astrocytes, and microglia. This notably resulted in improved hippocampal synaptic plasticity, improved amyloid plaque clearance by microglia, decreased tau phosphorylation, and decreased neuroinflammation (Varma et al., 2023). It should be noted that these effects required treatment to start before significant plaque accumulation. JAK inhibitors baricitinib and tofacitinib were both evaluated as possible treatments for AD, but they were found to have a low likelihood of success due to low BBB permeability (Faquetti et al., 2024). Baricitinib, however, is currently in an open-label, biomarker-driven basket trial in patients with subjective cognitive disorder, mild cognitive impairment, Alzheimer’s Disease (AD), Amyotrophic lateral sclerosis (ALS), or asymptomatic carriers of an ALS-related gene at Massachusetts General Hospital.

## 5 Conclusion

**Figure 1:**
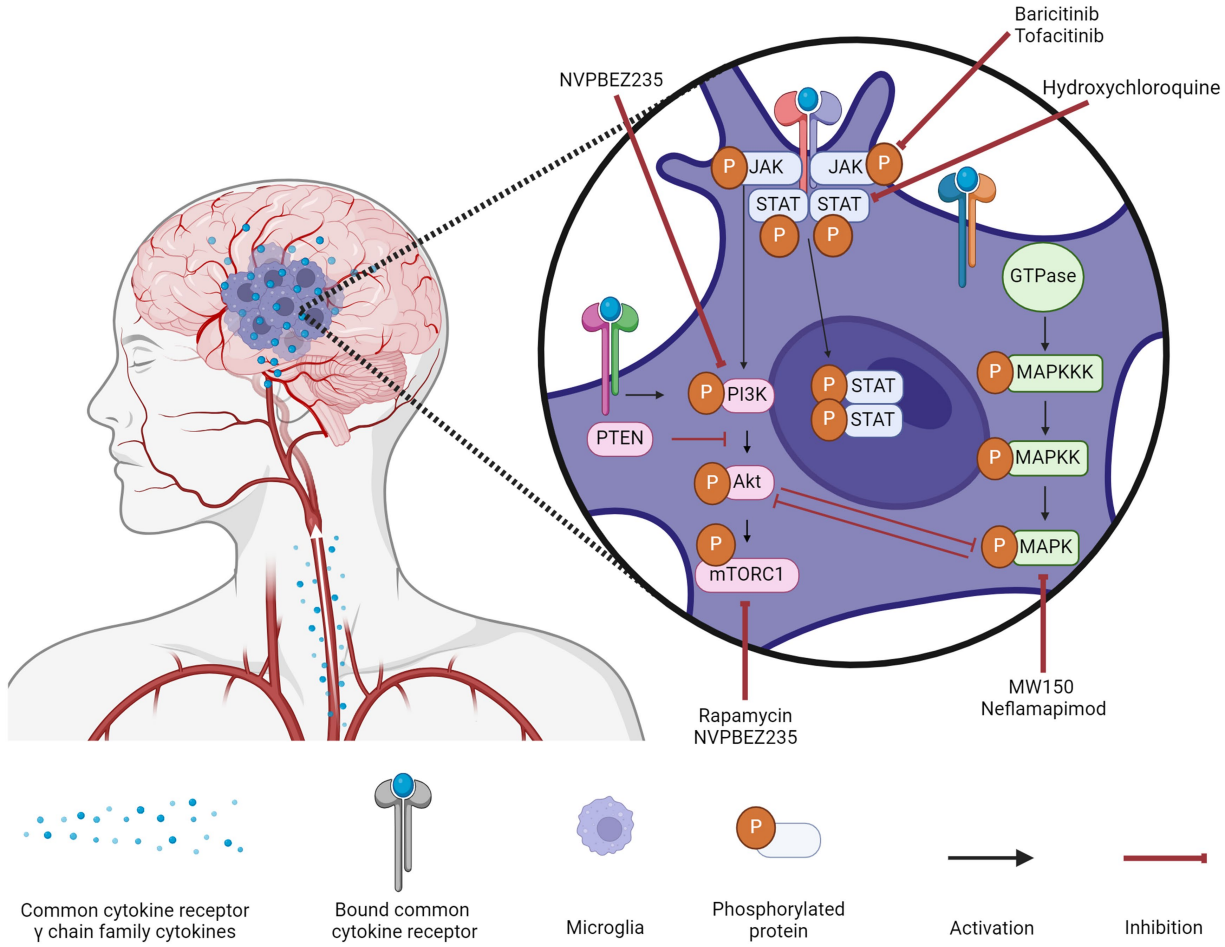
Microglial signaling cascades in Alzheimer’s disease and their inhibitors. Common cytokine receptor γ chain family cytokines activate three major signaling pathways (MAPK, PI3K, and JAK/STAT) in microglia during AD. A variety of inhibitors to components of these signaling cascades are being assessed for their efficacy in ameliorating the inflammatory milieu of the AD brain. Created with biorender.com.

The common cytokine receptor γ chain family of cytokines can activate three major signaling pathways in microglia to typically result in AD-promoting phenotypes (Figure 1). The MAPK pathway is associated with damaging inflammation, with all three MAPKs being notably altered in AD. The PI3K pathway is generally accepted as the central regulator of microglia activation in response to cytokines though there are conflicting results on the downstream effects of PI3K activation; it is unclear whether conflicting results are due to distinct signaling pathways being affected. The JAK/STAT pathway is responsible for quick signaling associated with pro-inflammatory responses. Common cytokine receptor γ chain family of cytokines typically activate JAK1 or JAK3 that then activate STAT1, STAT3, or STAT5.

The modulation of these three signaling pathways to treat AD (Figure 1) has been gaining traction though the clinical results are limited. Targeting the MAPK pathway can be difficult as upregulation of a specific kinase is not a guarantee that its inhibition will improve symptoms, as is the case with ERK kinases. Targeting the PI3K pathway could have the greatest effect given the numerous downstream targets, but for that same reason, it is also the most difficult to predict. While the backbone of the pathway is well-characterized, there is a lack of research into the downstream effects when different components of the core pathway are inhibited. Until these effects are identified, it will be difficult to transition from pre-clinical studies to clinical ones. Targeting the JAK/STAT pathway is popular due to FDA-approved JAK inhibitors being relatively safe and inexpensive, but low BBB permeability remains the greatest obstacle for this repurposing.

## Data availability statement

The original contributions presented in the study are included in the article/supplementary material, further inquiries can be directed to the corresponding author.

## Author contributions

HZ: Conceptualization, Writing – original draft, Writing – review & editing. ER: Funding acquisition, Supervision, Writing – review & editing.
